# Autosomal dominant polycystic kidney disease with ectopic unilateral multicystic dysplastic kidney

**DOI:** 10.1186/1471-2369-14-38

**Published:** 2013-02-17

**Authors:** Jing Xu, Dong-Ping Chen, Zhi-Guo Mao, He-Feng Huang, Chen-Ming Xu, Cong-Rong Wang, Wei-Ping Jia, Chang-Lin Mei

**Affiliations:** 1 Division of Nephrology, Kidney Institute of CPLA, Changzheng Hospital, Second Military Medical University, 415 Fengyang Rd, 200003, Shanghai, China; 2 Zhejiang University affiliated Gynecology and Obstetrics Hospital, Key Laboratory of Reproductive Genetics, Zhejiang University, Hangzhou, China; 3 Division of Endocrinology, Shanghai Diabetes Institute, Shanghai Sixth People’s Hospital, School of Medicine, Shanghai Jiao Tong University, Shanghai, China

**Keywords:** Autosomal dominant polycystic kidney disease, Ectopia, Multicystic dysplasia, Unilateral

## Abstract

**Background:**

Autosomal dominant polycystic kidney disease (ADPKD) is the most common hereditary renal disorder. In most cases, ADPKD similarly affects bilateral kidneys.

**Case presentation:**

Among the 605 ADPKD patients that were followed up by our center, we identified two male patients with unilateral ADPKD. The cases were remarkable because the patients also had ectopia and multicystic dysplasia in the contralateral kidney, which are generally sporadic disease conditions. Both patients tested positive for polycystic kidney disease 1 mutation, but negative for hepatocyte nuclear factor 1 beta mutation. Moreover, the deterioration of their kidney function seemed to be quicker than their age- and sex-matched controls and siblings. Both patients had started a long-term hemodialysis in their 40s.

**Conclusion:**

Anatomical and genetic abnormality in patients with ADPKD may be more frequent and complex than previously believed. The compensatory capacity in patients with ADPKD is fragile, and missing one kidney could accelerate the deterioration of renal function.

## Background

Autosomal dominant polycystic kidney disease (ADPKD) is the most common hereditary renal disorder and the fourth cause of death of end-stage renal disease, accounting for 10% of patients on dialysis [1]. The disease is caused by mutations in the *PKD1* (85% of cases) or *PKD2* (15% of cases) [2]. In most ADPKD patients, bilateral kidneys are similarly affected, with numerous fluid-filled cysts arising from different nephron segments. Only a few cases of ADPKD with unilateral renal agenesis or severe hypoplasia have been reported [2].

Among the 605 ADPKD patients followed by our center, we report two cases of ADPKD patients with pelvic ectopic unilateral multicystic dysplastic kidney (MCDK), which refers to a sporadic disease condition and is related to *transcription factor 2 (TCF2*) mutations [3]. The clinical characteristics of these two rare cases were compared with their siblings and other male ADPKD patients with similar ages; these characteristics were then summarized and analyzed. The *PKD1* and *PKD2* mutations and the relevant MCDK (*TCF2*) mutations were also measured.

## Case presentation

### Case 1

**Figure 1:**
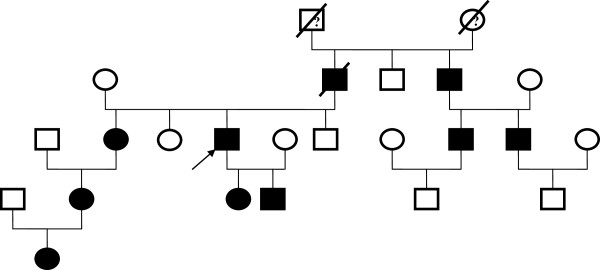
**Family tree of Case 1 patient.** (Arrow): affected proband with unilateral ADPKD; (filled square, filled circle): male/female affected by ADPKD with two kidneys; (blank square, blank circle): male/female did not have ADPKD; (?): unsure for ADPKD; (box with a stripe): family member passed away.

**Figure 2:**
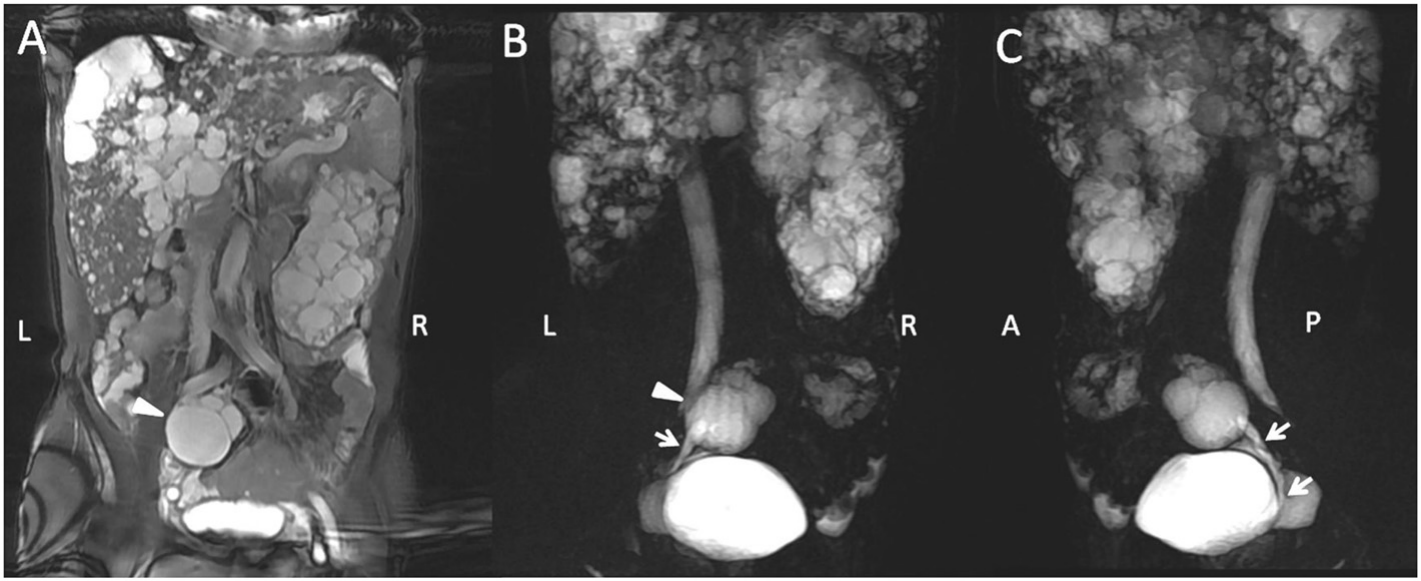
**MRI scans showing a solitary left polycystic kidney with contralateral MCDK in Case 1 patient.** (Triangle): right ectopic MCDK; (arrow): right ureter.

A 48-year-old man with ADPKD was admitted for long-term hemodialysis because his estimated glomerular filtration rate (eGFR) dropped to 14 ml/min/1.73 m^2^. His blood pressure was within the normal range. The patient was diagnosed with ADPKD 10 years earlier with a positive family history (Figure 1). He and his relatives had no history of intracranial aneurysm. He had no albuminuria and did not complain of renal pain. During diagnosis, an enlarged polycystic left kidney was found, but the right kidney was absent. An ultrasonography after admission detected a fist-sized, multi-cystic mass located before his right iliac artery. An enlarged polycystic liver was also detected. The magnetic resonance imaging (MRI) scan confirmed a 6 cm diameter polycystic mass in the right pelvis (Figure 2, Panels A and B, triangles), and the left kidney was significantly enlarged, measuring 18 cm × 12 cm × 8 cm. A renal volume assessment was then performed. The renal volume for the left kidney was 1127.6 cm^3^ and that for the right was only 74.6 cm^3^. Further MR urography revealed a tubular structure connecting the pelvic polycystic mass to the bladder with a fluid signal along the tubular lumen area (Figure 2, Panels B and C, arrows). This ADPKD patient had a right pelvic multicystic kidney with congenital aplasia, and the tubular structure was his right ureter.

### Case 2

**Figure 3:**
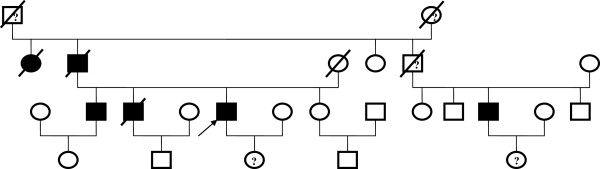
**Family tree of Case 2 patient.** (Arrow): affected proband with unilateral ADPKD; (filled square, filled circle): male/female affected by ADPKD with two kidneys; (blank square, blank circle): male/female not had ADPKD; (?): not sure for ADPKD; (box with a stripe): deceased family member.

**Figure 4:**
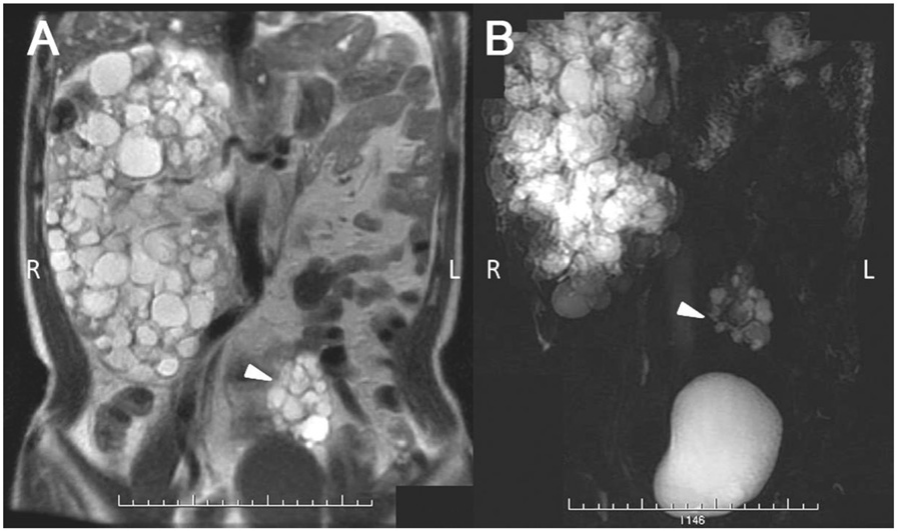
**MRI scans showing a solitary right polycystic kidney with contralateral MCDK in Case 2 patient.** (Triangle): left ectopic MCDK.

A 45-year-old male with fatigue and elevated creatinine (542 μmol/l, eGFR = 14 ml/min/1.73 m^2^) was admitted to set up long-term vascular access. The patient had hypertension controlled with nifedipine. No history of intracranial aneurysm was reported. He had a positive family history (Figure 3) and was diagnosed with ADPKD through an ultrasonography 16 years prior this study. He had no albuminuria and did not complain of renal pain. During diagnosis, an exaggerated right kidney was found. However, the left kidney was absent. After admission, MR urography detected a 22.8 cm × 12.2 cm × 10.5 cm right kidney with a typical polycystic phenotype, and a 5.8 cm × 4.2 cm × 3.9 cm multicystic space-occupying lesion before the left psoas (Figure 4, Panels A and B, triangles), with signals similar to the right polycystic kidney, indicating a small left pelvic kidney with multicystic aplasia. Based on renal volume assessment, the left kidney measured 2290.7 cm^3^, whereas the right one only measured 44.0 cm^3^. A polycystic liver was also detected. The CT scan result agreed with that of MR urography.

### Screening and genotyping

No ectopic aplastic cystic kidney was detected among the family members of these two patients, both affected and non-affected, who were screened using abdominal ultrasonography. These two patients were the only ones with this presentation among their families. A genetic analysis of *PKD1* and *PKD2* was performed by Athena Diagnostics (MA, U.S.A.) and verified at the Zhejiang University affiliated Gynecology and Obstetrics Hospital (Hangzhou, Zhejiang, China).

Genotyping of the two cases indicated that each had different mutations in the *PKD1* gene. Case 1 had a proline in place of leucine at amino acid 55 and a premature termination signal in codon 60 in exon 1 [NM_000296.3 P.L56PfsX60 (168_174dupCGCGGGC,novel,Het)]. Case 2 had a 7 bp duplication mutation (11650_11651) in exon 41, resulting in a frameshift at amino acid 3814, which is a predicted disease-associated mutation. He also had two nonsense mutations in exon 15 (an A in place of a G at nucleotide position of 6625) and exon 23 (an A in place of a G at nucleotide position of 8851). None of these polymorphisms had been previously reported.

Both patients tested negative for *TCF2* mutation, also known as *hepatocyte nuclear factor 1 beta* (*HNF1β*).

### Comparison of the reported cases to age- and sex-matched controls with bilateral ADPKD or siblings

A matched control group for each case was defined among the remaining 603 patients with ADPKD followed up by our center. All patients with the same sex and age (with no more than a five-year difference) from the respective ages of the cases were recruited. Three of their age- and sex-matched siblings were also chosen as another control group (Table 1). The single-kidney volume (SKV) and eGFR were expressed as mean and 95% confidence interval (CI). Compared with the matched bilateral ADPKD controls, the median SKV of the ectopic MCDK kidneys of the two reported cases were significantly smaller [Case 1: 74.6 vs. 692.4 95% CI (611.3, 781.3) cm^3^; Case 2: 44.0 vs. 657.1 95% CI (566.9, 743.4) cm^3^]. However, the SKV of their normal contralateral kidneys seemed to be significantly larger [Case 1: 1127.6 vs. 692.4 95% CI (611.3, 781.3) cm^3^; Case 2: 2290.7 vs. 657.1 95% CI (566.9, 743.4) cm^3^]. The mean eGFR of the two cases were significantly lower than their age- and sex-matched siblings (14.0 vs. 68.3, 39.6, 65.0 ml/min/1.73 m^2^) and bilateral ADPKD controls [Case 1: 14.0 vs. 84.7 95% CI (77.7, 91.7) ml/min/1.73 m^2^; Case 2: 14.0 vs. 88.8 95% CI (81.7, 96.4) ml/min/1.73 m^2^].

**Table 1:** Kidney volumes and renal function in two patients with ectopic unilateral MCDK compared with their age- and sex-matched siblings and bilateral ADPKD controls

**Parameter**	**Case 1**	**Age- and sex-matched bilateral ADPKD for case 1**	**Case 2**	**Age- and sex-matched bilateral ADPKD for case 2**	**Age- and sex-matched siblings**
No. of Patients	1	66	1	60	3
Age	48.0	47.9	45.0	45.6	41, 47, 49
Mean (range)		(43.2, 53.0)		(40.3, 49.7)	
SKV (cm^3^)	Left: 1127.6	692.4	Right: 2290.7	657.1	NA
Mean (95%CI)	Right: 74.6	(611.3, 781.3)	Left: 44.0	(566.9, 743.4)	
eGFR (ml/min/1.73 m^2^)	14.0	84.7	14.0	88.8	68.3, 39.6, 65.0
Mean (95%CI)		(77.7, 91.7)		(81.7, 96.4)	

*MCDK*: multicystic dysplastic kidney; *ADPKD*: autosomal dominant polycystic kidney disease; *SKV*: single-kidney volume; *eGFR*: estimated glomerular filtration rate; *NA*: not available.

## Discussion

In most cases, bilateral kidneys are involved in ADPKD patients. No previous report has been conducted on unilateral ADPKD with contralateral ectopic MCDK.

Among the 605 patients followed up by our center, we identified two subjects with unilateral ADPKD. No kidney tissue on the contralateral location was found on both patients’ kidneys. MRI revealed ectopic dysplasia kidney remnants with multiple cysts in the pelvis. Both patients had advanced disease during presentation. Compared with their matched bilateral ADPKD controls, the median SKV of the ectopic MCDK kidneys of the two reported cases were significantly smaller (Table 1), whereas their contralateral kidneys were significantly larger. The mean eGFR of the two cases (Table 1), which indicated a more advanced stage of disease progression with the unilateral ADPKD, were significantly lower than their age- and sex-matched siblings and bilateral ADPKD controls.

In a cohort of 182 patients with ADPKD, Poster et al. [2] identified three patients with unilateral renal agenesis or severe hypoplasia. These three cases had different truncating mutations in their *PKD1* gene. Although their kidney volumes and volume progression rates were greater than the mean values of their two polycystic kidney controls, which is normally associated with an accelerated decrease in renal function, the eGFR in these patients was remarkably well-preserved [4]. This characteristic may be partly due to compensatory parenchymal hypertrophy. However, renal parenchyma is unlikely to make a large contribution to the total kidney volume because the enlarged single cystic kidneys were grossly cystic, as shown in the MRI images [2]. Moreover, all their patients were much younger than ours (23 years old, 38 years old, and 40 years old), and the disease progressions of the three subjects after long-term follow-up remain unknown. The two patients in the present study had different mutations on the *PKD1* gene. The relationship between the *PKD1* mutation sites and the disease progression was difficult to explain because of the small sample size limitation of the reported cases.

Apart from unilateral ADPKD, our patients also had concurrent ectopic MCDK. MCDK generally refers to a sporadic disease condition of abnormal metanephric differentiation. The incidence is approximately 1 in 4300 in the general population [3]. We identified two cases of MCDK among the 605 ADPKD patients, indicating an incidence rate of 3.3%. Generally, the total kidney function of many subjects with MCDK could be well-maintained [5]. However, both our patients prepared for long-term hemodialysis when they were in their 40s, mainly in consideration of the kidney structure destruction by the continuous cyst growth in their contralateral kidneys.

*TCF2* (*HNF1β*) abnormalities cause congenital anomalies of the kidney and urinary tract [6]. Some studies have reported that MCDK is related to *TCF2* mutation [7,8]. However, both of our patients tested negative for *TCF2* mutation. Mutations in genes such as *EYA1*, *SIX1*, and *PAX2* are related to the occurrence of MCDK. However, neither of our patients had concomitant ear and eye structural abnormalities or dysfunction (data not shown). Their MCDK could be related to other genetic abnormalities.

## Conclusions

We conclude that the anatomical and genetic abnormality in patients with ADPKD could be more frequent and complex than previously believed. Patients with ADPKD have fragile compensatory capacities, and missing one kidney could accelerate the deterioration of the renal function.

## Consent

The clinical information of the two patients and their relatives were provided by the index patients or their relatives after obtaining their consents. The study was conducted following the Declaration of Helsinki. Written informed consents were obtained from the patients for publication of this case report and any accompanying images. Copies of the written consents are available for review by the editor of this journal.

## Abbreviations

ADPKD: Autosomal dominant polycystic kidney disease; CI: Confidence interval; eGFR: Estimated glomerular filtration rate; HNF1β: Hepatocyte nuclear factor 1 beta; MCDK: Multicystic dysplastic kidney; MRI: Magnetic resonance imaging; SKV: Single-kidney volume; TCF2: Transcription factor 2

## Competing interests

The authors declare that they have no competing interests.

## Authors’ contributions

XJ participated in the conception and design of the study, data acquisition, and data analysis and interpretation, performed the statistical analysis, and drafted the manuscript; CDP participated in the data acquisition and statistical analysis; MZG participated in the conception and design, as well as in data acquisition; HHF, XCM, WCR, and JWP carried out the genetic studies on mutations; MCL participated in the design and coordination and helped revise the study critically for important intellectual content. All authors read and approved the final manuscript.

## Pre-publication history

The pre-publication history for this paper can be accessed here:

http://www.biomedcentral.com/1471-2369/14/38/prepub
